# The Eye as an Aid in General Diagnosis

**Published:** 1897-09

**Authors:** 


### The Eye as an Aid in General Diagnosis. A handbook for the use of students and general practitioners. By E. H. Linnell, M. D., 618 Minor Street, Philadelphia: The Edwards & Docker Co., 1897. Cloth bound, $2.00.

This book, as is indicated by the name, is designed to afford the practitioner in general medicine an additional means of diagnosing diseases by taking advantage of the appearances afforded by the eye. These appearances "afford valuable aid not only in the diagnosis of diseases of the central nervous system, but also of constitutional affections and diseases of other organs."

The first chapter gives the affections of the external eye, lids, conjunctiva, orbit, and cornea.

•The second chapter deals with the ocular muscles for moving the ball of the eye. The peculiar affections of these muscles, and the diseases they indicate or suggest are given.

Affections of the lens and iris, and their relationship to general diseases are made the subject of the third chapter.

The fourth chapter deals with the appearances perceived by aid of the ophthalmoscope. The author thinks that every one should be familiar with the manipulation of the ophthalmoscope, and that it should be a common, every-day instrument in the hands of those who do not make a specialty of eye treatment. No physician should attempt to treat cases involving lesions of the kidneys without having the capacity to use the ophthalmoscope and the inclination to put it into service in detecting these affections of the renal glands.

The relation of retinitis albuminurica to pregnancy is specially discussed, and attention is also called to retinitis syphilitica, diabetica, and leukæmica. Gouty retinitis and "choked disc" are treated, and then special affections of the optic nerve are considered.

Chapter fifth discusses questions of sight, and in a more abbreviated way goes over the ground discussed in Dr. Ranney's book on *Eye-Strain*, which was reviewed in the August number of this journal.

The distribution of fibres in the two optic nerves and to either eye is discussed and the plan of these fibres from their origin in the cortex of the brain to their termination on the retina of each eye is vividly shown by a beautiful colored plate.

Finally there is a table consisting of a list of diseases with the eye-appearances, internal and external, occurring for each disease. This table is very good as far as it goes, but we suggest that there should be another chapter following this one in which the table is exactly reversed, the eye-appearances being placed in alphabetical order and the names of the diseases which they indicate being placed immediately after them. Such a table as this would be of very great service and very much enhance the value of the book. It is intended to be a standard book of reference and consultation, and this arrangement would at once establish its reputation.

In chapter seventh we have a discussion of the relation of ocular affections to functional nervous diseases, especially epilepsy, chorea, neurasthenia, and hysteria. This, also, is ground touched on by other authors, and particularly the one before quoted.

One of the most interesting parts of the book is the section devoted to the effect of toxic influences upon the eye. Of this class of influences attention is most held by the consideration of the effects respectively of whisky and tobacco. The effects of Quinine, Salicylate of Soda, Ergotine, Caffeine and other drugs are given, and then we have the effects of toxic agents not medicinal, as Aniline, Arsenic, Snake poison and Phosphorus.

Books like this one are, to the study of diseases and their diagnosis, what the *Repertory* is to the study of the materia medica—they are indispensable.

